# Chemical and Biological Evidence of the Efficacy of Shengxian Decoction for Treating Human Lung Adenocarcinoma

**DOI:** 10.3389/fonc.2022.849579

**Published:** 2022-03-18

**Authors:** Kejuan Li, Fengming You, Qin Zhang, Ruijiao Yuan, Qianghua Yuan, Xi Fu, Yifeng Ren, Qian Wang, Xiaohong Li, Zhenya Zhang, Mototada Shichiri, Yue Yu

**Affiliations:** ^1^ College of Life Science, Sichuan Normal University, Chengdu, China; ^2^ Traditional Chinese Medicine (TCM) Regulating Metabolic Diseases Key Laboratory of Sichuan Province, Hospital of Chengdu University of Traditional Chinese Medicine, Chengdu, China; ^3^ Oncology Department, Hospital of Chengdu University of Traditional Chinese Medicine, Chengdu, China; ^4^ Graduate School of Life and Environmental Sciences, University of Tsukuba, Tsukuba, Japan; ^5^ Biomedical Research Institute, National Institute of Advanced Industrial Science and Technology (AIST), Ikeda, Japan; ^6^ DBT-AIST International Laboratory for Advanced Biomedicine (DAILAB), AIST, Tsukuba, Japan

**Keywords:** Shengxian Decoction, Chinese medicine, lung cancer, anti-tumor agent, toxicity

## Abstract

Shengxian Decoction (SXT) is a traditional Chinese medicine prescription comprising several anti-cancer medicinal herbs. However, the anti-cancer effect of SXT has rarely been reported. Herein, we explored the therapeutic potential of SXT for the treatment of lung adenocarcinoma (LUAD). High-performance liquid chromatography analysis of crude SXT extract revealed the abundance of mangiferin, an established anti-cancer compound. The serum pharmacological evaluation revealed that serum SXT suppressed A549 lung cancer cell proliferation *in vitro*. The tumor-inhibitory activity of SXT was confirmed *in vivo via* tumor formation assays in nude mice. We applied biochemical, histopathological and imaging approaches to investigate the cellular targets of SXT. The results indicated that the treatment with SXT induced tumor necrosis, and downregulated hypoxia-inducible factor 1 alpha in the serum. *In vivo* biosafety assessment of SXT revealed low levels of toxicity in mouse models. Our study provides the first scientific evidence that SXT effectively represses cancer cell growth and, thus, may serve as a safe anti-cancer agent for LUAD treatment.

## Introduction

Lung adenocarcinoma (LUAD), a histological subtype of non-small cell lung cancer, is one of the most common malignancies in terms of morbidity and mortality, accounting for approximately 40% of lung malignancies (1). Due to the characteristics of insidious onset, rapid metastasis and high recurrence rate, LUAD is usually first diagnosed at advanced stages. The five-year overall survival rate of LUAD patients is < 25% (2). LUAD can currently be treated with surgical resection, cytotoxic drug therapy, thoracic radiotherapy, targeted therapy, immunotherapy or a combination of these techniques (3). However, recurrence remains highly likely. The complete cure rate is < 10% (4). Although immunotherapy and targeted therapy provide new directions for improving the survival of patients with LUAD, the cost is generally exorbitant. Therefore, the search for new and cheaper drug candidates for LUAD treatment has become the focus of current research.

Shengxian Decoction (SXT), a well-known traditional Chinese formula, comprises Astragali Radix (A. radix, 18 g), Anemarrhenae Rhizoma (A. rhizoma, 9 g), Bupleuri Radix (B. radix, 4.5 g), Platycodonis Radix (P. radix, 4.5 g) and Cimicifugae Rhizoma (C. rhizoma, 3 g). (5). SXT can be used to treat experimental autoimmune myasthenia gravis (6), cardiomyocyte injuries (7), and chronic heart failure (8). The anti-cancer activity of SXT has rarely been reported; however, the individual constituents of SXT have been shown to attenuate cancer growth. For example, as the main component in SXT, A. radix inhibited LUAD development through regulation of the autophagy process (9). Meanwhile, B. radix and P. radix have been shown to have favorable anti-cancer effects in various cancer models, including human LUAD (10–13). Moreover, C. rhizoma and A. rhizoma exhibit strong cytotoxicity against human breast and colorectal cancer cells (14–16). Previous phytochemical investigations revealed three types of flavonoids (i.e. mangiferin, calycosin-7-O-β-D-glucoside and formononetin) in A. radix (17, 18), isoferulic acid in C. rhizoma (19), Timosaponin AIII in A. rhizoma (20), saikosaponins in B. radix (11), and Platycodin D in P. radix (13). These compounds are believed to be the major active components that account for their corresponding anti-cancer effects. Hence, we hypothesized that their integrated formulation (SXT) may have a therapeutic effect on LUAD.

**Figure 1:**
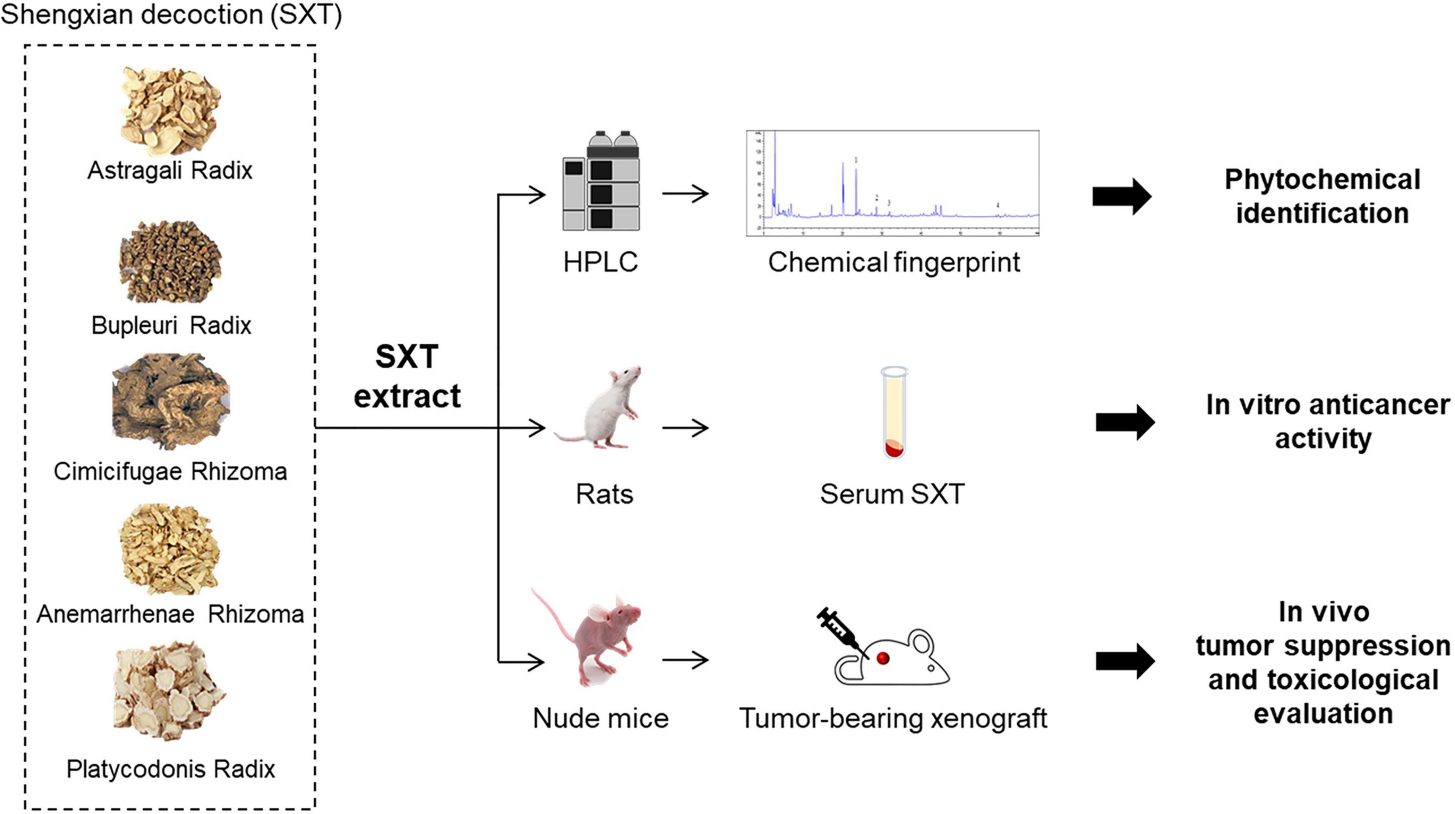
Schematic illustration of the experiment design in this study. Shengxian Decoction (SXT) extracted from five medicinal herbs was subjected to high-performance liquid chromatography (HPLC) analysis for phytochemical identification, followed by an integrative serum pharmacology-based approach to evaluate cancer killing activity *in vitro*. The *in vivo* anti-tumor effect of SXT and preliminary toxicological evaluation was investigated using mice.

Serum pharmacology is a novel method that can be used to realistically analyse pharmacological actions of drugs after their metabolization in the body. In serum pharmacology, drug or drug combinations are orally administrated to animals, and the blood from treated animals is subsequently collected after a certain period to separate the drug-containing serum for further experimental studies (21). Serum pharmacology is regarded as a reliable approach for evaluating the anti-tumor activities of traditional Chinese medicine (22). Therefore, to explore the possibility of SXT as a candidate drug for LUAD treatment, we used serum pharmacology method, together with a subcutaneous xenograft model, to study the anti-cancer effect of SXT on A549 human LUAD cells (**Figure 1**). High-performance liquid chromatography (HPLC) was also employed to identify potential phytochemicals in SXT (**Figure 1**).

## Materials and Methods

### Medicines and Chemicals

A. radix (Gansu, China, batch No. 1901032), A. rhizoma (Hebei, China, batch No. 190301), B. radix (Hebei, China, batch No. 190401), P. radix (Anhui, China, batch No. 1904065), and C. rhizoma (Liaoning, China, batch No. 1902051) were provided by the Affiliated Hospital of Chengdu University of Traditional Chinese Medicine. Mangiferin (batch No. 190822, 98.56%), calycosin-7-O-β-D-glucoside (batch No. 190817, 99.21%), formononetin (batch No. 191206, 98.61%) and isoferulic acid (batch No. 191115, 98.06%) were purchased from Chengdu Plant Standard Pure Biotechnology Co., Ltd. (Sichuan, China). Isotonic sodium chloride solution (0.9% saline) was purchased from Sichuan Kelun Pharmaceutical Co., Ltd. (Sichuan, China, batch No. A19042103-1). Cisplatin (Cis-diamminedichloroplatinum, DDP) was purchased from Jiangsu Hausen Pharmaceutical Co., Ltd. (Sichuan, China, batch No. 190602).

### Cell Culture

The human LUAD cell lines A549, SK-LU-1 and NCl-H1975 were obtained from the American Type Culture Collection. A549 cells were maintained in Dulbecco Modified Eagle Medium (Life Technologies, CA, USA), supplemented with 10% fetal bovine serum (FBS) and 1% (v/v) penicillin-streptomycin. SK-LU-1 cells were maintained in Minima Essential medium (Life Technologies, CA, USA) supplemented with 10% FBS and 1% (v/v) penicillin-streptomycin. NCl-H1975 cells were maintained in Roswell Park Memorial Institute 1640 (Life Technologies, CA, USA) medium supplemented with 10% FBS and 1% (v/v) penicillin-streptomycin. The cells were incubated in a stable environment with 5% CO_2_/95% air at 37°C in a humidified incubator.

### Animals

Sprague-Dawley rats (male, 6-week old, 200 ± 20 g) for the serum pharmacology experiment were purchased from Chengdu Dasuo Experimental Animal Co., Ltd. (Sichuan, China). BALB/c-nude mice (6-week old, 20 ± 2 g) for the *in vivo* tumor suppression assay were purchased from Beijing Weitong Lihua Laboratory Animal Technology Co., Ltd. (Beijing, China). Animals were acclimated to the facilities for 5 days prior to the experiments in a specific pathogen-free environment, housed under controlled conditions (22 ± 3°C, approximately 60% humidity, and 12 h light-dark cycle), and fed with sterilized water and standard rodent chow. All feed was provided by Chengdu Dasuo Laboratory Animal Co., Ltd. All animal experiments were conducted in accordance with strict ethical guidelines, following the recommendations of the Animal Experiments Committee of Chengdu University of Traditional Chinese Medicine.

### Preparation of Crude SXT Extract

A total of 39 g (A. radix, 18 g; A. rhizoma, 9 g; B. radix, 4.5 g; P. radix, 4.5 g; C. rhizoma, 3 g) crude powders were weighed and soaked in eight times the volume of water (312 mL) for 2 h prior to the first decoction. The decoction was filtrated after boiling for 30 minutes, and the residues were collected for two additional decoctions under the same conditions. The total decoctions combined from three separate boiling instances were concentrated under reduced pressure (0.08 - 0.09 MPa) at 70°C. The resulting concentrate was subjected to freeze-drying until getting lyophilized powders (14.43 g; 37% extraction rate). SXT extract was prepared in double distilled water before use and stored at 4°C.

### SXT Dose Calculation

SXT doses used in this study were estimated using the factor method, in which the conversion factor for human/rat and human/mice were determined as 6.3 and 9.1, respectively, based on the body surface area (23). In the traditional Chinese medicine system, 39 g of SXT crude powder is clinically used for preparing the decoction that is usually administrated to humans with average body weight around 70 kg. According to the extraction rate (37% of SXT extract obtained from initial crude powder), we determined the clinical dose of SXT as 0.21 g/kg/d (39 g/70 kg/d × 37%) for human. Therefore, the equivalent dose for a 200 g Sprague–Dawley rat will be 6.3 × 0.21 kg/d = 1.32 g/kg/d. SXT doses used in the serum pharmacological experiments for rats were set as 5 times (6.60 g/kg/d), 15 times (19.80 g/kg/d) and 20 times (26.40 g/kg/d) of the calculated equivalent doses. The SXT dose for mice (1.91 g/kg/d) was obtained in a similar manner except for the conversion factor of 6.3 was replaced with 9.1. After calculation, the low, medium and high doses of SXT used in the *in vivo* anti-tumor assay were set as 9.55 g/kg/d, 28.65 g/kg/d, and 38.20 g/kg/d, respectively. The experimental doses of DDP were 15.48 mg/kg/d and 22.36 mg/kg/d for rats and mice based on its clinical dose (2.46 mg/kg/d), respectively.

### Preparation of Drug-Containing Serums

After adaptive feeding, the Sprague–Dawley rats were randomly divided into five groups (n = 6) and subjected to following treatments: normal saline (negative control, oral gavage), DDP (positive control, 15.48 mg/kg/d, intraperitoneal injection), low-dose SXT extract (6.60 g/kg/d, oral gavage), medium-dose SXT extract (19.80 g/kg/d, oral gavage), and high-dose SXT extract (26.40 g/kg/d, oral gavage). Each rat was administered twice a day with an interval of 12 hours.

On day 7, rat serum was collected as follows: the rats were anaesthetised by intraperitoneal injection of 10% chloral hydrate. Blood was then collected from the abdominal aorta and allowed to stand for 2 h. After centrifugation at 3000 rpm/min for 15 min at 4°C, the supernatant in the total blood was filtered through a 0.22 μm filter and placed in a 56°C water bath for 30 min to remove complements. The resulting drug-containing serums corresponding to normal saline, DDP, low-dose SXT, medium-dose SXT, and high-dose SXT treatments were labeled as Normal-S, DDP-S, LSXT-S, MSXT-S and HSXT-S, respectively, and was stored at -20°C until further use.

### Cell Viability Assay

A Cell Counting Kit-8 (Dojindo Laboratories, Kumamoto, Japan) was used to assess LUAD cell proliferation. Briefly, 100 μL of cell suspension (1 × 10^5^ cells/mL) was seeded in a 96-well plate and allowed to adhere overnight. Next, they were incubated with culture medium containing 10 μL of Normal-S, DDP-S, LSXT-S, MSXT-S or HSXT-S. After 24 or 48 h of treatment, 10 μL of Cell Counting Kit-8 solution was added to each well and incubated at 37°C for 4 h. Cell viability was assessed according to the change in absorbance at 450 nm using a microplate reader (Model 550; Bio-Rad, CA, USA).

### *In Vivo* Anti-Tumor Assay

Mice bearing A549-derived tumors were generated by subcutaneously injecting A549 cells (1 × 10^7^ cells in 0.2 mL medium) into the right axilla region. Tumor growth was monitored every second day. Successfully established tumor-bearing models were randomly divided into five groups (n=5) and administration was initiated as follows: saline (vehicle control, oral gavage); DDP (positive control, 22.36 mg/kg/d, intraperitoneal injection); low-dose SXT (LSXT) (9.55 g/kg/d, oral gavage), medium-dose SXT (MSXT) (28.65 g/kg/d, oral gavage), and high-dose SXT (HSXT) (38.20 g/kg/d, oral gavage). All the groups received treatment for 24 consecutive days. Body weight and tumor size were measured every two days. The tumor volume was calculated according to the formula: V (mm^3^) = 0.5× L × S^2^, where L and S are the long and short diameters of the tumor, respectively (24). Blood from each animal was collected from the eyeball at the end of the experiment for further analysis. Major organs including lung, spleen and tumor were isolated, pictured, weighed and sampled for further use. The tumor inhibitory rate was calculated using the following equation: inhibition rate (%) = (W*c* - W*e*) × 100/W*c*, where W*c* is the tumor weight of the control mice, and W*e* is the mean weight of the treated mice. Lung and spleen indices were calculated according to the following equation: Organ index (mg/g) = weight of organ (mg)/body weight (g) (25).

### Histopathological Evaluation

Tumors, lungs and spleens were fixed in 10% neutral buffered formalin, embedded in paraffin, and sectioned at a thickness of 4 μm. H&E staining was conducted according to routine protocols (26). Briefly, representative sections were stained with hemotoxylin and eosin, and examined using a digital microphotography system (BA200, Motic China Group Co., Ltd, Xiamen, China). The lesions in each section were imaged at magnifications of 100×, 200× or 400×. The necrosis region was analyzed using the ImageJ software. At least three fields of view were collected to calculate the quantitative result.

### Measurement of Biochemical Parameters in Blood

After collection, blood samples from each mouse were allowed to stand at room temperature for 2 h, and then centrifuged at 3000 rpm for 15 min at 4°C to obtain the serum. The levels of tumor necrosis factor-alpha (TNF-α) and hypoxia-inducible factor 1-alpha (HIF-1α) in the serum were measured using enzyme-linked immunosorbent assay (ELISA) kits from ZCIBIO Technology Co., Ltd (Shanghai, China) according to the manufacturer’s instructions. In Brief, 50 μL of serum samples were loaded into the wells, followed by the addition of 100 μL horseradish peroxidase-linked antibodies. After incubation at 37°C for 1 h, the samples were washed and then reacted with 50 μL substrates for 15 min. Absorbance of each well at 450 nm was assessed using a microplate reader (SpectraMAX Plus384, MD, USA). TNF-α and HIF-1α levels were calculated according to the calibration curves.

### HPLC Analysis

SXT extract was diluted with methanol to obtain a solution at a concentration of 72.15 mg/mL. Standard solutions of mangiferin (0.2328 mg/mL), calycosin-7-O-β-D-glucoside (0.0339 mg/mL), isoferulic acid (0.0470 mg/mL) and formononetin (0.0154 mg/mL) were carefully prepared with methanol by stepwise dilution of the stock solutions. All samples were filtered through a 0.22 -μm filter prior to HPLC injection.

Measurement was performed on an HPLC equipment (Agilent 1260 equipped with DAD detector) with an Agilent SB C18 column (250 × 4.6 mm, 5 µm; Agilent, CA, USA), and data were collected and analyzed using Agilent ChemStation software. Column temperature was maintained at 25°C. The mobile phase was composed of a 0.2% phosphoric acid solution (solvent A) and acetonitrile (solvent B) with gradient elution for better separation. Gradient solvent system was optimized as follows: 0-20 min, 1-17% B; 20-25 min, 17-20% B; 25-33 min, 20-23% B; 33-40 min, 23-30% B; 40-50 min, 30-33% B; 50-65 min, 33-50% B; 65-70 min, 50-70% B at a flow rate of 1.0 mL/min. The detection was conducted at 220 nm with a 5 μL injection volume of each sample.

### Statistical Analysis

All data were analyzed using the SPSS 26.0 software and the GraphPad Prism 8 software. Results were expressed as mean ± standard deviation (SD), and statistical significance was evaluated using analysis of variance (ANOVA) followed by a multiple comparison test with Duncan’s adjustment. Statistical significance was set at P < 0.05.

## Results

### Identification of Anti-Cancer Compounds in SXT Extract

**Figure 2:**
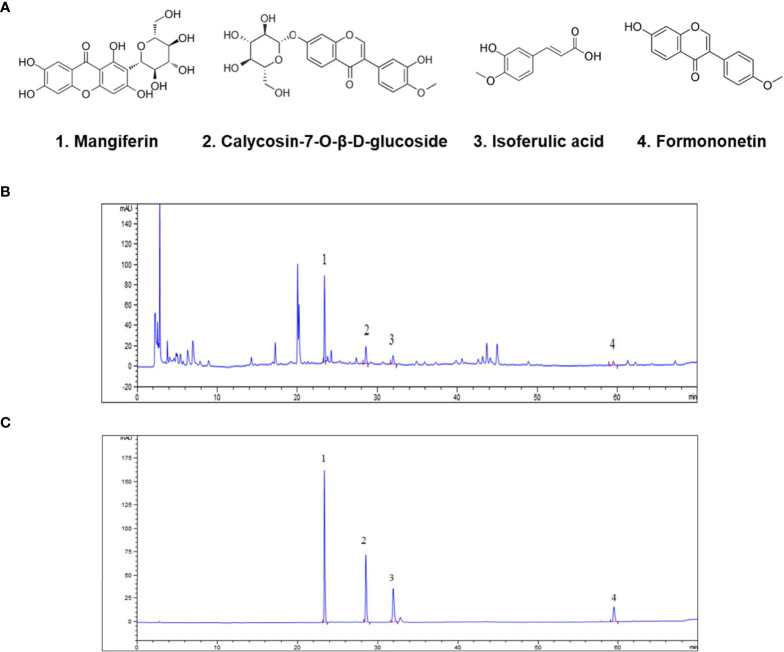
Identification of anti-cancer phytochemicals in SXT by HPLC analysis. **(A)** Potential chemical compounds in SXT responsible for anticancer effect. HPLC chromatograms of SXT extract **(B)** and standard sample mixtures of the potential compounds **(C)**. Peak assignment as follows: 1. mangiferin; 2. calycosin-7-O-β-D-glucoside; 3. isoferulic acid; 4. formononetin.

The chemical fingerprint of the SXT extract was determined using HPLC analysis. We selected four chemicals possessing anticancer properties as standard samples for comparison with the SXT extract (**Figure 2A**). The analysis results of SXT showed a number of peaks (**Figure 2B**), indicating the presence of multiple substances. Comparing with the standards (**Figure 2C**), compounds corresponding to peaks 1-4 were identified as mangiferin, calycosin-7-O-β-D-glucoside, isoferulic acid and formononetin, respectively. The contents of these compounds in SXT are listed in **Table 1**. These results indicate that the prepared SXT extract contains substantial amounts of anti-cancer compounds and may be capable of killing cancer cells.

**Table 1:** Amounts of bioactive compounds in SXT.

Compounds	mg/g ^1^
mangiferin	2.03 ± 0.08
calycosin-7-O-β-D-glucoside	0.16 ± 0.01
isoferulic acid	0.21 ± 0.002
formononetin	0.07 ± 0.003

Results are expressed as mean ± SD (n=3 independent measurements).

^1^Calculated mass in the SXT extract powder.

### Serum SXT Suppresses the Proliferation of LUAD Cells *in Vitro*

**Figure 3:**
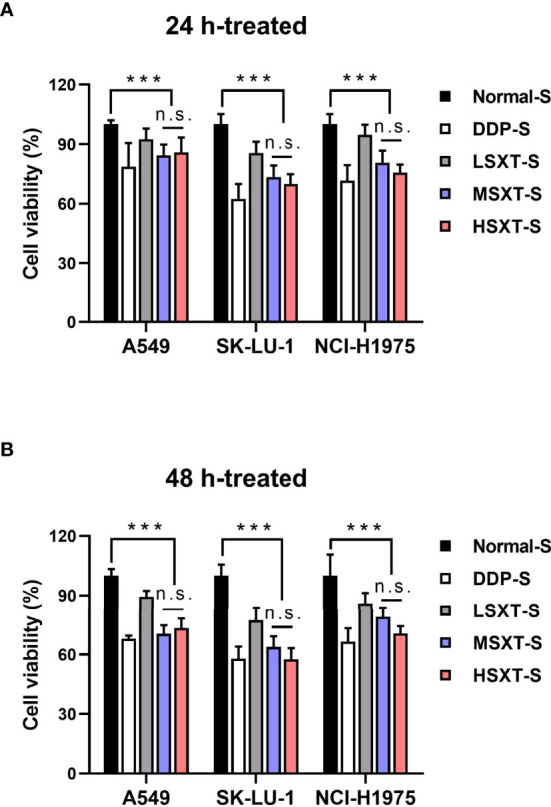
Serum SXT suppresses the proliferation of lung adenocarcinoma cells. Incubation with serum SXT for **(A)** 24 h and **(B)** 48 h show obvious cytotoxicity to lung adenocarcinoma cell lines (mean ± S.D., n=10), ***P < 0.001, n.s., no significance (one-way ANOVA test). Normal-S, normal serum; DDP-S, serum containing cisplatin; LSXT-S, serum containing low-dose SXT; MSXT-S, serum containing medium-dose SXT; HSXT-S, serum containing high-dose SXT.

Next, we performed a serum pharmacology assay to investigate the anti-cancer potential of SXT. To collect SXT-containing serum, Sprague–Dawley rats were administered crude SXT extract at different concentrations (see Material and Methods). The obtained serums corresponding to high, medium, and low doses of SXT were named HSXT-S, MSXT-S and LSXT-S, respectively. The *in vitro* cytotoxicity of SXT-S was evaluated using Cell Counting Kit-8 kit after 24 or 48 h of incubation with LUAD cells. DDP-S was used as a positive control. As shown in **Figure 3**, compared to that of the Normal-S control group, SXT-S treatments at all doses showed cytotoxic effects against A549, SK-LU-1, and NCl-H1975 cells. Notably, the cell killing performances of MSXT-S and HSXT-S were comparable to that of the DDP-S positive control, indicating favorable anti-tumor activity. However, the viability of cells treated with HSXT-S showed no significant difference (P > 0.05) compared to that of MSXT-S both at 24 and 48 h, which is likely a result of the similar amount of SXT presented in MSXT-S and HSXT-S.

### SXT Extract Retards the Tumor Growth *in Vivo*

**Figure 4:**
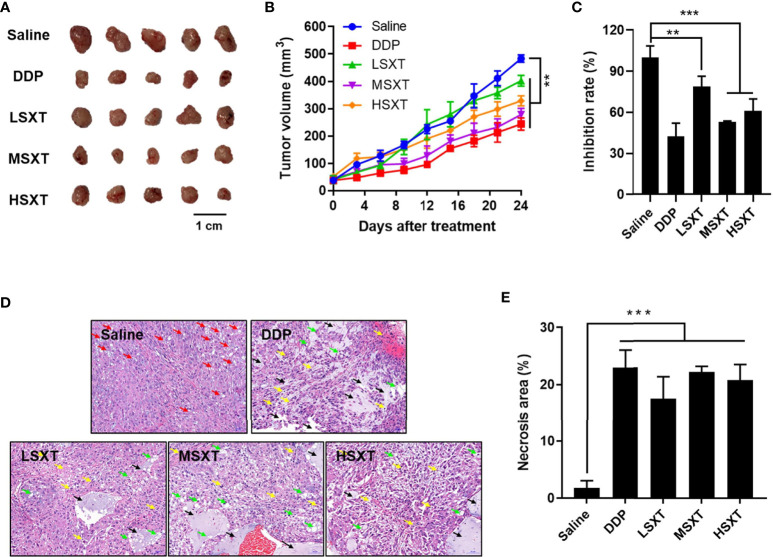
*In vivo* anti-tumor effect of SXT. **(A)** Image of tumors isolated from mice treated with 0.9% saline (vehicle control), 22.36 mg/kg/d of cisplatin (DDP, positive control) and 9.55 g/kg/d (LSXT), 28.65 g/kg/d (MSXT) and 38.20 g/kg/d (HSXT) of SXT extract after 24 days of treatment. **(B)** Tumor volumes in different groups of mice during treatment (measured at Day 0, 4, 8, 12, 24) (mean ± S.D., n = 5), **P < 0.01 (two-way ANOVA test). Subcutaneous injection of A549 cells in SXT -treated nude mice showed low tumor forming capacity as compared to that treated with the saline control. **(C)** Inhibition rate of A549 xenograft (in weight) in mice with different treatments at the end of experiment (mean ± S.D., n = 5), **P < 0.01, ***P < 0.001 (one-way ANOVA test to saline). **(D)** Representative histological images tumor sections stained with haematoxylin and eosin showing the induction of damages and necrosis after SXT treatment at different concentrations. Objective magnification: 200×. Red arrow: lipid droplets; Green arrow: nucleus fragmentation; Yellow arrow: vacuolar degeneration; Black arrow: necrosis. **(E)** The quantitation result is shown on the right (n = 3). ***P < 0.001 (one-way ANOVA test to saline).

Considering the promising anti-proliferation results *in vitro*, we next performed *in vivo* assays to investigate the effect of SXT on tumor progression in mice. A549 subcutaneous xenograft-bearing mice were administered saline, DDP, and different doses of SXT extract for 24 days. Visible differences in tumor size were observed among the groups by the end of treatment (**Figure 4A**). We found that the mice treated with saline exhibited aggressive tumor growth, with an average volume of 482.99 ± 13.52 mm^3^ at the end of experiment (**Figure 4B**). In contrast, tumors in STX-treated groups grew slower over time, of which volumes on day 24 were only 402.38 ± 12.70 mm^3^, 278.43 ± 23.15 mm^3^, and 328.65 ± 18.62 mm^3^ for LSXT (9.55 g/kg/d), MSXT (28.65 g/kg/d) and HSXT (38.20 g/kg/d) groups, respectively (**Figure 4B**). The tumors were excised from the mice and weighed to calculate the tumor inhibition rate. Notably, the results of MSXT were comparable to DDP positive control with a tumor inhibition rate of approximately 46.85% (**Figure 4C**). These data indicate that SXT is capable of retarding tumor progression *in vivo*. The encouraging therapeutic effect was highly consistent with *in vitro* observations.

To further investigate the anti-cancer activity of SXT, the isolated tumors were subjected to histological examination. As shown in **Figure 4D**, tumor tissues in the vehicle control group (saline) have a dense structure on a large scale, with intact nuclei and circular lipid droplets (as indicated by the red arrow). No obvious necrotic areas were observed. In contrast, tumors that received SXT treatments exhibited low cell density and irregular cell shape. Damage, such as nuclear fragmentation (green arrow), vacuolar degeneration (yellow arrow), and necrosis (black arrow), were also observed. Quantitation results (**Figure 4E**) revealed approximately 24.35%, 22.18%, 19.51% and 16.35% increases in tumor necrosis region in response to DDP, MSXT, HSXT, and LSXT treatments, respectively.

### SXT Reduces the Levels of TNF-α and HIF-1α in Mice Blood Serum

**Figure 5:**
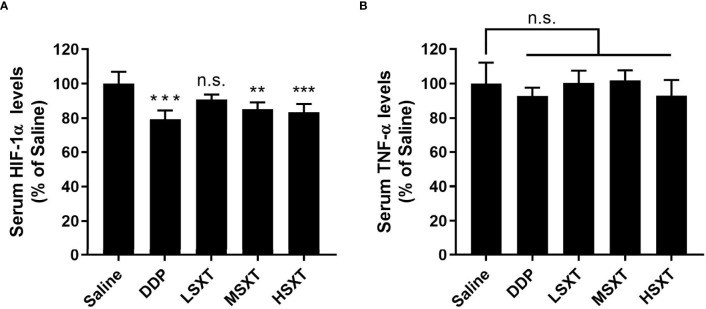
Relative expression levels of serum HIF-1α **(A)** and TNF-α **(B)** determined by ELISA (mean ± S.D., n = 5), **P < 0.01, ***P < 0.001, n.s., no significance (one-way ANOVA test to saline). SXT treatment at medium (MSXT; 28.65 g/kg/d) and high (HSXT; 38.20 g/kg/d) doses decreased the levels of HIF-1α, but not TNF-α. DDP was used as a positive control.

HIF-1α plays a key role in the development of cancer. Elevated HIF-1α levels are associated with tumor metastasis, poor patient prognosis and drug resistance (27). HIF-1α expression is positively regulated by TNF-α (28), a major inflammatory cytokine that induces necrosis in certain tumor types (29). Therefore, we evaluated their levels in the blood of mice following SXT treatment. As shown in **Figure 5A**, compared to the control mice injected with saline, HIF-1α levels were significantly downregulated, after 24 days of exposure to the medium (MSXT) and high (HSXT) doses of SXT. Quantitation results revealed 20.63%, 14.67% and 16.49% reductions in the expression levels of HIF-1α in the DDP (control drug), MSTX, and HSTX groups, respectively (**Figure 5A**). However, TNF-α levels showed no changes in all treatment groups (**Figure 5B**), indicating that SXT-mediated tumor necrosis is involved in TNF-α regulation.

### Evaluation of Biosafety Potentials of SXT Using Mice

**Figure 6:**
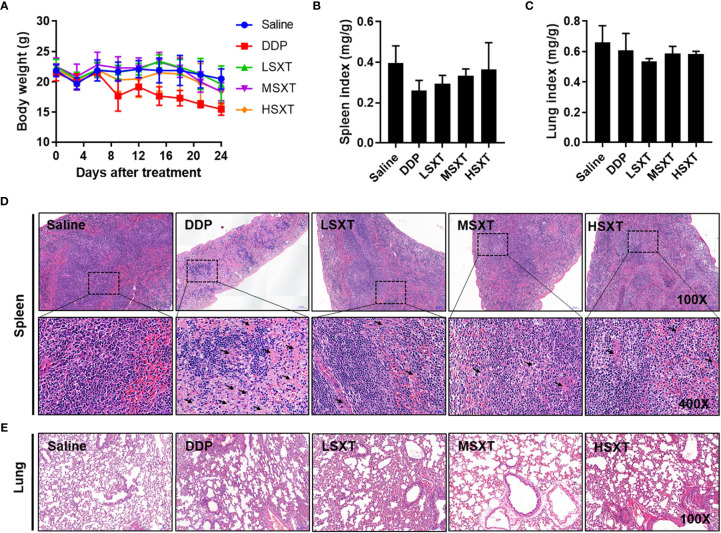
Toxicological evaluation of SXT. **(A)** Average body weight of mice showing no loss during treatments. Spleen **(B)** and lung **(C)** indices of mice following administrations of 0.9% saline, DDP (22.36 mg/kg/d) and low (9.55 g/kg/d; LSXT), medium (28.65 g/kg/d; MSXT) and high (38.20 g/kg/d; HSXT) doses of SXT extract (mean ± S.D., n = 5). The data indicates no significant difference between SXT and saline control groups. **(D)** Histological images (magnification: original 100×; enlarged 400×) of spleen in treated mice. Treatment with commercial drug DDP induces pathological changes in the spleen tissue, whereas SXT exhibits no sign of toxicity. Black arrow indicates the germinal centre. **(E)** Observation of lung histopathology showing no abnormal changes at the tested doses of all the treatments.

Multiple herbal medicines are pharmacologically beneficial at one dose, however, they can be toxic at another (30). The safety of traditional medicines remains a concern. Therefore, we evaluated the biosafety potential of SXT at the various therapeutic doses used in our study. Administration of SXT as high as 38.20 g/kg/d (HSXT) showed negligible changes in mice body weight over the treatment period (**Figure 6A**). Notably, we observed a noticeable reduction in body weight in the mice treated with the commercial drug, DDP. However, MSXT, whose anti-cancer effect is comparable to that of DDP (**Figure 4**), exhibited no significant loss in body weight (**Figure 6A**), demonstrating that SXT could potentially be a potent medication with low toxicity.

The effect of SXT on organ toxicity was also assessed by measuring organ indices (organ weight/body weight). We selected the spleen and lung as representative organs because their injuries frequently occur during chemotherapy (31, 32). As shown in **Figures 6B, C**, neither spleen nor lung indices exhibited significant differences (P > 0.05) between the control and treated groups. Saline-treated mice showed a normal splenic tissue structure, as evidenced by the intact dorsal membrane and clear boundaries between white (in blue) and red (in red) pulps (**Figure 6D**). In contrast, DDP treatment resulted in splenic atrophy, lymphocyte reduction, formation of germinal centre, sparsely arranged red marrow cells, and enhanced light reduced cell density. Compared with that of the DDP group, SXT-treated mice showed moderate pathological changes in the spleen tissue structure. Only a slight degree of this phenomenon was observed (**Figure 6D**). No obvious histological changes were observed in the lung tissue in any of the groups (**Figure 6E**), suggesting that SXT did not impair the lung. Altogether, these results demonstrated that SXT, within the concentration of 38.20 g/kg/d, caused limited organ damage in mice.

## Discussion

Increasing evidence suggests that traditional Chinese medicine is beneficial for improving the clinical outcomes of LUAD patients (10). SXT has been widely used for treating diseases related to immune and cardiovascular diseases in China for a long time (6–8). Although the therapeutic use of SXT for cancer treatment has not been extensively studied, its constituent herbs have been shown to be useful for the treatment of cancers, including LUAD (10, 12, 15, 16, 33). For instance, A. radix, as the main component of SXT, has been recently reported to kill lung cancer cells by regulating the p53/AMPK/mTOR signaling pathway (9). B. radix and P. radix, as auxiliary prescriptions in SXT, have also been shown to induce apoptosis in cancer cells (11, 13). We verified that SXT is effective in inhibiting lung cancer growth both *in vitro* and *in vivo*.

Earlier reports revealed that mangiferin, calycosin-7-O-β-D-glucoside, and formononetin might be the major active components responible for the anti-cancer effects of A. radix (17, 18). Isoferulic acid is believed to be responsible for the colorectal cancer-killing activity of C. rhizoma. (19). Among them, mangiferin. exhibits anti−tumor properties in A549 xenograft mice *in vivo*, and may negatively regulate the expression of miR-92a and miR-27b to influence not only cancerous growth but also the cell cycle progression and apoptosis induction of LUAD cells (34, 35). Formononetin inhibits tumor growth by suppression of EGFR-Akt-Mcl-1 axis in non-small cell lung cancer (36). Therefore, we examined the potential anti-cancer constituents (mangiferin, calycosin-7-O-β-D-glucoside, formononetin and iso-ferulic acid) in SXT by HPLC. The results showed that SXT is abundant in mangiferin (0.203%), which may account for the favorable tumor suppression performance of SXT.

The *in vitro* anti-proliferation activity of SXT on LUAD cells (A549, SK-LU-1, and NCl-H1975) was investigated using serum pharmacology experiments. The results indicated that SXT had anticancer effect comparable to that of DDP (in the case of MSXT-S). Furthermore, an *in vivo* tumor suppression assay suggested that administration of SXT for 24 days repressed tumor progression with inhibition rates of 21.16%, 46.85% and 38.96% (**Figure 4C**) for LSXT, MSXT and HSXT, respectively. These encouraging therapeutic effects are highly consistent with *in vitro* observations.

LUAD development is a complex biological process involving numerous factors, and the activation of inflammatory cytokines is one of the most important incentives. TNF-α is a major inflammatory cytokine, and has been reported to play decisive roles in the complex process of lung cancer onset, progression, and dissemination (37). Furthermore, the malignancy level of LUAD is closely related to the tumor microenvironment, where cancer cells use glycolysis more readily as a metabolic pathway for energy metabolism (38). HIF-1α, which plays a regulatory role in glycolytic metabolism, is a core regulator of restoring intracellular environmental stability under hypoxia (39), and functions in the control of cell proliferation (22). Stabilization of HIF-1α in LUAD promotes glycolysis, thereby enhances tumor metastasis (5). Using ELISA technique, we detected a significant decrease in HIF-1α levels in the blood serum of mice receiving SXT treatment (**Figure 5**) suggesting, at least in part, that SXT may exert anti-tumor activity by modulating HIF-1α-related signaling pathways.

Observation of mouse body weight revealed hardly any toxicity of SXT at all doses used in the present study. Since spleen is an important organ that functions in blood cell storage and filtration, its index is regarded as an acceptable indicator of immune response (40). As shown in **Figure 6B**, no significant difference in spleen index was observed, indicating that SXT hardly impaired spleen-related immune capacity in mice. In addition, treatment with SXT and DDP, had no influence the on the lung tissue structure (**Figure 6C**).

In conclusion, the data presented herein demonstrate SXT is effective in LUAD treatment, with low toxicity to visceral tissues. The therapeutic performance of SXT (at a medium dose, 28.65 g/kg/d) was comparable to that of the commercial anticancer drug DDP; however, it was considerably safer. Our study provides an experimental basis for developing new anticancer agents from SXT for the treatment of LUAD.

## Data Availability Statement

The original contributions presented in the study are included in the article/supplementary material. Further inquiries can be directed to the corresponding author.

## Ethics Statement

The animal study was reviewed and approved by Animal Experiments Committee of Chengdu University of Traditional Chinese Medicine.

## Author Contributions

KL, FY and YY conceived and designed the experiments. KL, QZ, RY, QY, XF, YR, QW and XL. performed the experiments. KL, ZZ, MS and YY analyzed the data. KL, FY and YY prepared the draft. All authors discussed the results and contributed in writing the manuscript.

## Funding

KL thanks for the financial support from the National Natural Science Fund of China (81904081). YY is grateful to the Japan Society for the Promotion of Science (JSPS) KAKENHI Grant-in-Aid for Early-Career Scientists (21K14508). DAILAB is funded by DBT (Government of India) and AIST (Japan).

## Conflict of Interest

The authors declare that the research was conducted in the absence of any commercial or financial relationships that could be construed as a potential conflict of interest.

## Publisher’s Note

All claims expressed in this article are solely those of the authors and do not necessarily represent those of their affiliated organizations, or those of the publisher, the editors and the reviewers. Any product that may be evaluated in this article, or claim that may be made by its manufacturer, is not guaranteed or endorsed by the publisher.
